# Tailored thermal emission in bulk calcite through optic axis reorientation

**DOI:** 10.1515/nanoph-2023-0005

**Published:** 2023-05-12

**Authors:** Katja Diaz-Granados, Weiliang Ma, Guanyu Lu, Joseph Matson, Peining Li, Joshua D. Caldwell

**Affiliations:** Interdisciplinary Materials Science, Vanderbilt University, Nashville, TN 37212, USA; Wuhan National Laboratory for Optoelectronics and School of Optical and Electronic Information, Huazhong University of Science and Technology, Wuhan 430074, China; Department of Mechanical Engineering, Vanderbilt University, Nashville, TN 37212, USA

**Keywords:** crystal anisotropy, far-field emissivity, tilted optic axis

## Abstract

The polar nature of calcite results in lattice vibrations that can be stimulated through gratings and nanostructures to design spatially and spectrally coherent thermal radiation patterns. In order to obtain optimal design control over such patterned materials, it is first necessary to understand the fundamental emissivity properties of the lattice vibrations themselves. Because calcite is a uniaxial material, when the optic axis (OA) is tilted with respect to the crystal surface, the surface wave solutions to Maxwell’s equations and vibrational modes that are permitted will change due to the crystal’s structural anisotropy. This implies that the OA orientation can play a critical role in dictating which modes can be harnessed when designing a narrowband or angular thermal emitter. Here we explore the angle and polarization dependence of the bulk far-field emissivity of unpatterned calcite with tilted OA. We show that by manipulating the OA orientation via crystallographic off-cut, polarization, and sample rotation, the emissivity at a given frequency can vary by as much as 0.8. These results suggest that, in addition to serving as a basis for modifying the behavior of the relevant phonon polaritons, OA orientation can be used to alter the thermal emission pattern without the need for complex lithographic patterning.

## 1 Introduction

The uniaxial crystal calcite (CaCO_3_) has traditionally found use in optics as a component in retardation plates and polarizing prisms, where its strong birefringence enables polarization control in the visible and near-infrared. In the context of far-field thermal emission, manipulating the orientation of the optic axis (OA) could offer a way to design emissivity patterns using bulk low-symmetry crystals [1–3] with multiple cleavage planes, either eliminating the need for diffractive outcoupling through patterned gratings [4], nanostructures [5–9], layered heterostructures [10], and superlattices [11], or enhancing the complexity that can be achieved with such altered surfaces. In this paper, we provide an experimental demonstration of the multiple degrees of freedom with which the OA orientation can be manipulated in unpatterned calcite to modify the resultant thermal emission spectra and radiation patterns.

We demonstrate that reorienting the OA through crystallographic off-cut, polarization, and sample rotation corresponds to a modulation of the far-field emission. Using mechanical cleavage to decrease the angle between the OA and surface normal, we can achieve reduced emissivity for the in-plane modes coincident with enhanced emissivity for the out-of-plane modes. We see that the same effect can also be achieved by rotating an individual sample or by restricting the emitted radiation to a specific polarization, with vibrational peaks again appearing or disappearing at any fixed frequency. Detailing how the change in sampled modes translates into a far-field thermal emission signature will enable simple bulk crystals to be used for tailoring radiation patterns and emission spectra, as well as establish a point of departure for the creation of more complex designs incorporating nanostructures or gratings. The ability to define asymmetric thermal emission patterns in this way has potential use for the control of spontaneous emission [12, 13], thermophotovoltaic systems [14], pattern-free thermal modulators [15], and near-field radiative heat transfer [14, 16, 17].

## 2 Results

**Figure 1:**
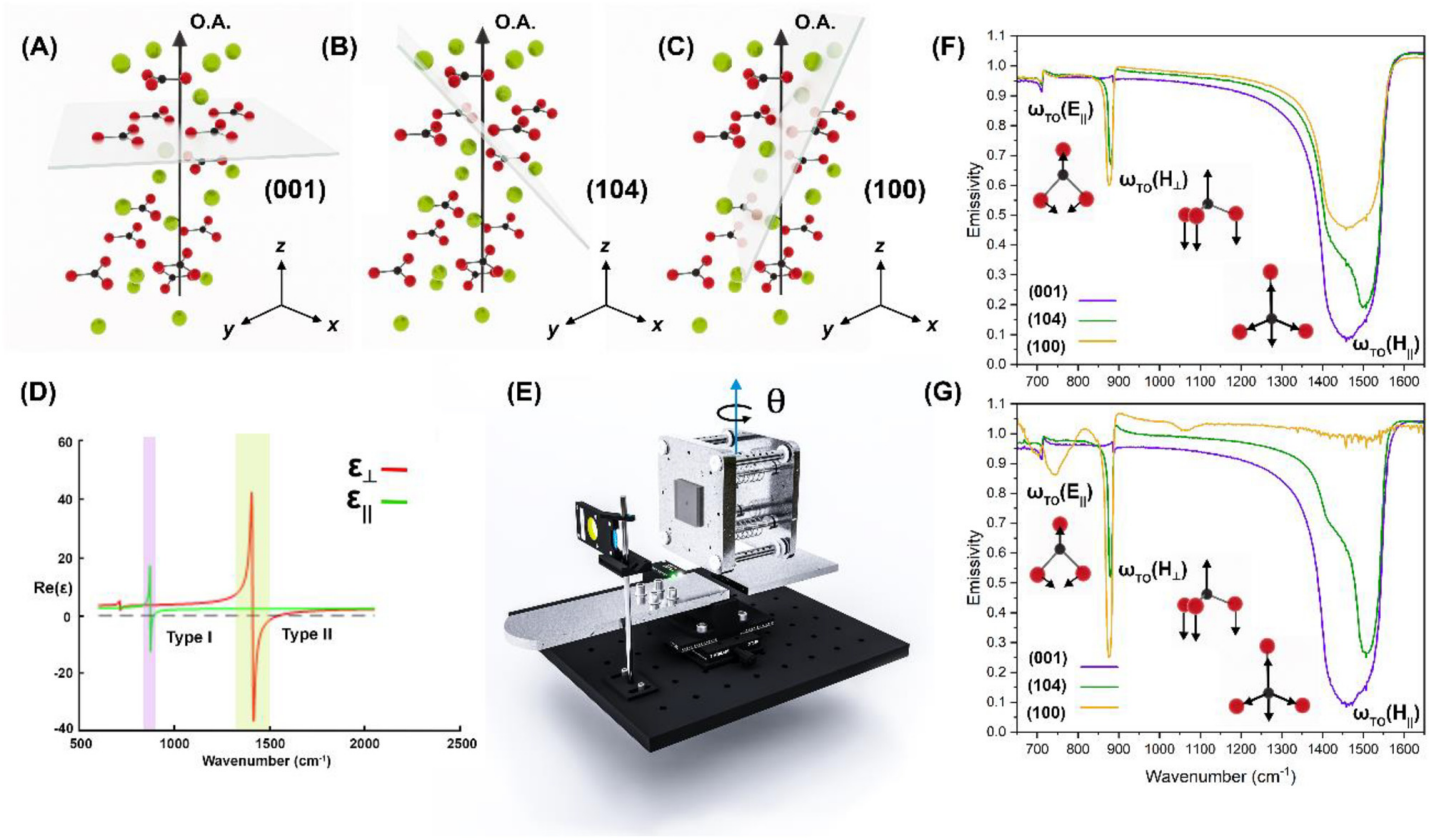
Experimental set-up and comparison of three OA tilt angles. (a)–(c) Schematic of a calcite unit cell with different cleavage planes (green for calcium, black for carbon and red for oxygen). Crystal structure model imported from Avogadro [20]. (d) Real part of the parallel and perpendicular permittivity components of the dielectric function with shaded regions for Type I and Type II Reststrahlen bands. (e) Illustration of thermal emission rotation rig with a sample mounted onto the heating face. (f) 0° polarization and (g) 90° polarization thermal emission for three tilt angles of the crystal off-cuts. Insets show vibrational modes.

To study the asymmetric emission from bulk calcite crystals, the vibrational normal modes with differing OA configurations were examined. Each unit cell of calcite contains two formula units of CaCO_3_ that are arranged as an alternating stacking of planar CO_3_^2−^ and Ca^2+^ ions, with calcium octahedrally coordinated with oxygen (Figure 1(a)–(c)). Of the 27 vibrational normal modes, those where Ca^2+^ and the CO_3_^2−^ experience translations relative to one another lie at lower frequencies than the vibrations of CO_3_^2−^ alone. This is due to the difference in strength between the ionic bonding between Ca^2+^ and CO_3_^2−^ and the intramolecular bonding within the CO_3_^2−^. The Ca^2+^ and CO_3_^2−^ lattice modes all fall below 400 cm^−1^ and are therefore outside our spectral range of interest. We instead focus solely on those intramolecular modes that are infrared active. These primary vibrations of calcite are labeled *ω*_TO_(*H*‖), *ω*_TO_(*H*⊥), and *ω*_TO_(*E*‖), with *E* and *H* referring to elliptical and hyperbolic modes (with Type I and Type II hyperbolic regions distinguished in Figure 1(d)), respectively, and ‖ and ⊥ indicating whether the vibration involves an in-plane or out-of-plane motion (insets in Figure 1(f) and (g)). The *ω*_TO_(*H*⊥) mode is a transverse optic (TO) phonon characterized as a predominantly out-of-plane bending (A_2u_) and is found at 871 cm^−1^, with the matched longitudinal optic (LO) phonon at 890 cm^−1^. The spectral region between these two modes is referred to as a Reststrahlen band (RB) [18]. A second RB extends from ω_TO_(*H*||) at 1410 cm^−1^ (TO) to its paired LO at 1550 cm^−1^, with the former being a phonon driven by an in-plane asymmetric stretch (*E*_u_). The final RB of interest begins at *ω*_TO_(*E*‖), which appears as an in-plane bending action of the C–O bond (*E*_u_) and is located at 712 cm^−1^ (TO) with its pair at 715 cm^−1^ (LO). It is within these RBs that phonon polaritons [19], and thus, the potential for coherent emissivity patterns, can be observed.

Each of the three primary vibrational modes represents an electric dipole offset of the carbonate ion from its equilibrium position. The specificity of these vibrations is what allows infrared spectroscopy to be used to glean information about the material properties of a crystal. In particular, the characteristic vibration frequency will be informed by all aspects of the bond responsible for creating the dipole offset. In those materials in which there is pronounced structural anisotropy, the orientation of the chemical bonds becomes particularly important, as the orientation of the electric field and the chemical bond must match for the vibration to be incited. When such a match occurs, the energy from the propagating wave component can be subsumed by the vibrating dipole, and a dip will occur in the frequency dependent emissivity spectra, with the amount of radiated energy that is absorbed by the vibrating dipole depending on both the strength of the bond [21–23] and how well the orientation of the electric field and chemical bond match.

Thermal emissivity can also be directly related to optical absorption **— **and, by extension, reflection and transmission measurements **— **via Kirchoff’s law. This law states that *ɛ*(*ω*, *T*, *ψ*, Φ) = *A*(*ω*, *T*, *ψ*, Φ), where *ɛ* and *A* represent the emissivity and absorptivity, respectively, with both being functions of the incident frequency, *ω*, temperature, *T*, polarization state, *ψ*, and the angle relative to the surface normal, Φ. If the influence of scattering upon the extinction is disregarded, the fundamental additive nature of light interactions dictates that 1 = *R*(*ω*) + *T*(*ω*) + *A*(*ω*). This correspondence between reflectivity, transmissivity, absorptivity, and emissivity is crucial for engineering thermal emission, as it allows for predictions of which spectral regions will have high or low emissivity based on their reflectivity and absorptivity. In the case of a polar crystal like calcite, for the frequencies that constitute RBs, we would expect high reflectivity, and corresponding low emissivity, when the vibrations are being sampled. Pure calcite has an average emissivity near 1 [21, 24] due to its lossy nature across much of the infrared. The general spectral shape is thus characterized by a high emissivity across the thermal infrared region, punctuated by narrow dips in emissivity attributable to the vibrations of the C–O bonds.

In this work we use far-field emissivity measurements to identify changes in the spectra that arise from the orientation of the OA. Our chosen samples represent three distinct crystal off-cuts of calcite where the OA tilt angles are commensurate with those explored in the context of the ghost polariton investigations [25]. These were confirmed by XRD to have (001), (104), and (100) crystal faces, with each sample being 1 cm × 1 cm and 3 mm thick and single-side polished. Through the choice of crystallographic off-cut, polarization, and sample rotation, we explore how the excited vibrational modes depend on the OA orientation in both the in-plane and out-of-plane directions. The tilt angle of the OA effectively corresponds to an out-of-plane rotation, with the three off-cuts representing three different angles of rotation (Figure 1(a)–(c)). To understand the effect of the in-plane orientation of the OA, a wire grid polarizer was placed in front of the sample and the collected thermal emission was linearly polarized (Figure 1(e)). Finally, to capture rotation about an emission angle (*θ*), each sample was secured to a rotating heating stage that was used to control the angle of the sample surface with respect to the collection optics in a home-built variable angle thermal emission rig (Figure 1(e)) [9]. For all of the thermal emission measurements, the sample was heated to, and held at, a temperature of 300° C.

The first sample configurations explored are those where the angle between the OA and the sample surface was changed, for which the three different crystal off-cuts were used as representative samples. For the (001) sample, the OA is aligned parallel with the surface normal, with the carbonate ions defined to lie in the plane perpendicular to the OA (Figure 1(a)). The (104) (Figure 1(b)) and (100) (Figure 1(c)) samples are cut so that the OA is tilted away from the surface normal, with the (100) sample representing one extreme and the (104) sample representing an intermediate case. As the angle between the electric field vector and the OA increases, the ‖ and ⊥ modes follow opposing trends. The emission spectra for (001) registers lower emissivity values for *ω*_TO_(*H*‖) and *ω*_TO_(*E*‖) modes, while the *ω*_TO_(*H*⊥) mode is at its highest in this off-cut (Figure 1(f) and (g)). The (104) off-cut has an intermediate emissivity compared to the (001) and (100) off-cuts. With the intermediate tilt angle of the (104) sample, the *ω*_TO_(*H*⊥) mode decreases and the *ω*_TO_(*H*‖) and *ω*_TO_(*E*‖) modes both increase (Figure 1(f) and (g)). Having the exposed surface of the crystal cut so that carbonate ions are oriented partially out of the plane means that vibrations of both the in-plane ( $\|\left.\right)$ and out-of-plane $\left(⊥\right)$ types are sampled. Because the carbonate ions are no longer in a flat plane, the in-plane modes both change, in the sense that less of the emitted energy is diverted by the vibrating molecules and the resulting drop in emissivity is less drastic. When the crystal is cut along the (100) plane, so that the OA is close to aligned with the surface plane, the *ω*_TO_(*H*⊥) mode continues to decrease and the *ω*_TO_(*H*‖) and *ω*_TO_(*E*‖) modes both increase, though in the case of the 90° polarized spectra the *ω*_TO_(*E*‖) is similar in intensity to what is measured for the (104) sample (Figure 1(f) and (g)).

By changing between the three crystal off-cuts, there seems to be an overall trend where reducing the angle between the OA and surface normal allows for a decrease in the emissivity of the in-plane modes and an increase in the emissivity of the out-of-plane modes. The magnitude of change that can be affected is relatively large, even with the choice of only three off-cuts. Notably, at a frequency of 1450 cm^−1^ (*ω*_TO_(*H*‖)), a change in 90° polarized emissivity from 0.995 for (100) to 0.099 for (001) was observed, while for a frequency of 875 cm^−1^ (*ω*_TO_(*H*⊥)), we see a change from 0.255 for (100) to 0.963 for (001).

Changing the cleavage plane appears to be the equivalent of choosing the degree to which the in-plane versus out-of-plane atomic motion is sampled. When the crystal is cut so that the carbonate ions are lying flat within the surface plane, the in-plane modes at *ω*_TO_(*E*‖) and *ω*_TO_(*H*‖) form well-defined dips in intensity, while the one out-of-plane mode at $\omega_{\text{TO}}\left(H⊥\right)$ is not sampled (leading to an almost flat spectral lineshape). By choosing crystal off-cuts where the OA is tilted to some degree away from the surface normal, the surface of the crystal will no longer have a full complement of carbonate ions that can be sampled. Rather, the carbonate ions will lie partially out of the plane, and so their sampled vibrational character will contain both the in-plane and out-of-plane components.

**Figure 2:**
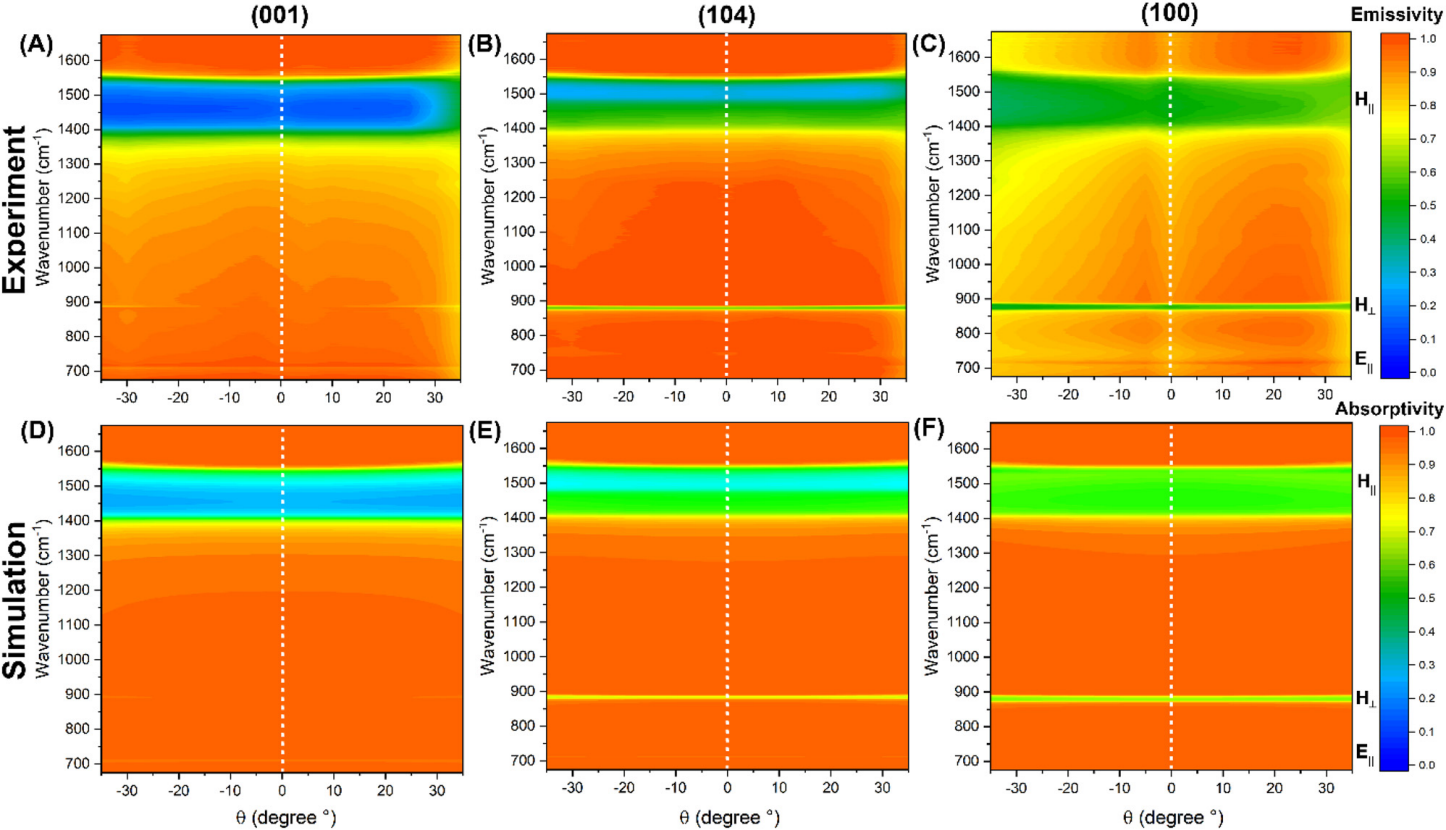
Experimental contour plots of unpolarized emissivity for (001) (a), (104) (b), and (100) (c) samples, measured at 5° angle increments. Simulated contour plots of unpolarized absorptivity for (001) (d), (104) (e), and (100) (f) samples, calculated for 5° angle increments. Data for both interpolated in Origin Labs.

If instead of comparing three distinct samples, an individual sample is rotated about *θ*, we observe that this also prompts contributions from vibrational modes based on their in-plane versus out-of-plane character with respect to the surface orientation. By manipulating *θ,* we are able to begin sampling the out-of-plane *ω*_TO_(*H*⊥) that was almost completely absent for the normal incidence (001) sample. Now, for the (001) surface, when the stage has not been rotated, the carbonate ions remain parallel to the surface plane (Figure 1(c)), such that both the *ω*_TO_(*H*‖) and *ω*_TO_(*E*‖) in-plane modes are sampled, while no drop in emissivity is seen for the *ω*_TO_(*H*⊥) out-of-plane phonon (Figure 2(a)). As the stage is rotated, however, the *ω*_TO_(*H*⊥) appears and its emissivity progressively decreases with increasing rotation angle (Figure 2(a)), suggesting that an increasing proportion of the extraordinary axis is contributing to the measured emissivity. This is consistent with the *E*‖*z* polarization selection rules associated with the *ω*_TO_(*H*⊥) mode. For the two in-plane modes of the (001) sample, there is relatively little change with rotation, with the exception of a slight increase in the experimentally measured emissivity with increasing positive *θ* angle. This can perhaps be attributed to a misalignment in the axis of rotation as we approached steeper angles, which may have resulted in a shift in the measurement position on the sample. A distinct behavior is observed for the (104) and (100) off-cuts, where the carbonate ions are canted relative to the surface plane (Figure 1(b) and (c)). For these samples, the emissivity profiles appear relatively insensitive to *θ*, with distinct bands of low emissivity spanning the range of angles (Figure 2(b) and (c)). This is not unexpected, as both the (104) and the (100) samples represent cases where the carbonate ions already lie partially tilted relative to the crystal surface. If the range of *θ* angles is broadened (Figure S4), however, there does seem to be a change in the emissivity profile that remains consistent with the in-plane and out-of-plane nature of the studied phonon modes in all three off-cuts. Due to the constraints of our experimental system, we performed numerical simulations based on the 4 × 4 transfer matrix method, which calculates the reflection and transmission coefficients of layered materials based on their dielectric tensors, to validate our experimental results [26]. With the exception of the noted discrepancy for Figure 2(a), we see that the absorptivity contour plots produced by the transfer matrix method generally agree with the experimental emissivity contour plots (Figure 2(d)–(f)).

The observed changes in the emissivity profiles can be explained as they relate to the Brewster effect. In the context of light reflecting at the boundary between a crystal and air, the intensity of reflected light at a particular incidence angle can drop to zero due to impedance matching [27]. This angle is called the Brewster angle, and it can be leveraged to create conditions for perfect absorption [28, 29] or to select for polarization (because the *p*-polarized component of the light will disappear). By rotating the heating stage about *θ*, we are effectively changing the ‘incidence’ angle of the radiated light. For this reason, we expect to see a change in the magnitude of absorptivity/emissivity that corresponds with the Brewster angle as we sample different rotations. Again, the additive nature of light implies that the absence of reflectivity will correspond to a high value of absorptivity/emissivity. Previous studies looking at the effect of the OA tilt angle on the optical properties of uniaxial crystals have pointed out that the angle at which the Brewster effect occurs will shift when the OA is tilted and that the occurrence of the Brewster effect follows opposing trends for Type I and Type II hyperbolic modes [30]. This is consistent with what we observe for the three calcite off-cuts. For the *ω*_TO_(*H*‖) mode, which is Type II hyperbolic and involves an in-plane motion, the (001) off-cut shows a strong band of low absorptivity/emissivity across rotation angles, with the intensity dropping as the *θ* angle increases (Figure S4). The very low absorptivity/emissivity (and consequently high reflectivity) implies that the Brewster effect does not play a role for the (001) off-cut. The progressive tilt angles of the (104) and (100) off-cuts, however, show more complicated patterns of absorptivity/emissivity within the *ω*_TO_(*H*‖) band (Figure S4). Since we are looking at unpolarized light, we expect the Brewster angle to correspond with a high measure of absorptivity, but not an absorption of 1 as would be the case for *p*-polarized light. Thus, as a general trend, higher emissivity (which we can attribute to the Brewster effect) seems to appear at smaller *θ* angles as the tilt angle of the OA decreases. For the *ω*_TO_(*H*⊥) mode, which is Type I hyperbolic and involves an out-of-plane motion, the opposite trend is seen, with there being high absorptivity/emissivity even at relatively high *θ* angles for the (001) off-cut, and with the transition from high to low emissivity occurring at progressively smaller *θ* angles for the (104) and (100) off-cuts (Figure S4).

**Figure 3:**
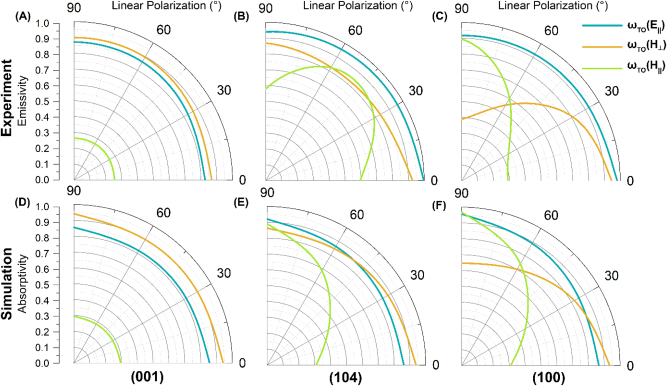
Measured polarization dependence of three primary vibration modes for (001) (a), (104) (b), and (100) (c). Transfer matrix method calculations of the polarization dependence for (001) (d), (104) (e), and (100) (f).

To understand the effect of the in-plane orientation of the OA, a wire grid polarizer was placed in front of the sample and the collected thermal emission was linearly polarized, with the rotation stage fixed at *θ* = 0° (Figure 1(e)). All the samples are placed in the same orientation; accordingly, the in-plane polarization angle is referred to as the angle of the polarizer. For the (001) orientation, the spectra changed only minimally with the polarization angle (Figure 3(a)). In this case, 0° and 30° polarizations led to a modest suppression of the emissivity across the entire spectral range. The 60° and 90° polarization conditions produced spectra with similar intensities. The fact that the three modes are relatively insensitive to polarization in the (001) sample is in keeping with the orientation of the IR active dipoles in this off-cut. Given that the carbonate ions lie within the surface plane, it follows that changing the in-plane polarization will have little to no effect on the sampled vibrations.

As with the previous manipulations to the OA orientation, we expect to see the vibrational modes that vibrate parallel to the OA to behave differently from the vibrational modes that vibrate perpendicular to the OA when the in-plane polarization angle is changed. For the (104) sample, the emissivity for the *ω*_TO_(*H*‖) mode first increases then decreases with increasing polarization angle (Figure 3(b)). The drop in emissivity at 0° and 90° polarization is a consequence of the peak shape, which takes on a slight shoulder at these two polarizations (as seen as well in Figure 1(f) and (g)). The shoulder at 0° and 90° polarization has been explained as arising from the polarization field splitting the *ω*_TO_(*H*‖) mode into two orthogonally polarized dipole moments [31]. Each carbonate ion is arranged in the surface, or *x*–*y*, plane and the symmetric stretch associated with the *E*_u_ mode is restricted to that plane. From this perspective, the displacement vector along the *y* direction will define TO phonons as expected, but the displacement vector along the *x* direction will be complicated by the tilted OA, which establishes an offset in the *x* axis relative to the surface normal of the crystal. If the light is polarized parallel to the wavevector, the LO phonons will create an electric field that acts upon the *x* component of the displacement vector. Regardless of the polarization of light, the *y* axis is fixed to be orthogonal to the surface normal and thus will experience no change. The result is that for light polarized parallel to the wavevector, the tilt in the OA will shift the low frequency side of the *ω*_TO_(*H*‖) band to both higher frequency and emissivity values. For the *ω*_TO_(*H*⊥) mode, the opposite trend is observed, with the emissivity being the lowest for 30° and 60° polarization, and emissivity increasing for the highest polarization angles of 0° and 90° (Figure 3(b)). The degree of linear polarization results were plotted using the TO frequencies of each mode. The fact that the shape of the simulated results in Figure 3(e) differs from our experimental findings could suggest that the dielectric function we used was not a perfect fit at that frequency.

From the (100) sample we see that selecting for specific linear polarizations of the emitted radiation is an effective way of excluding certain modes from being sampled. In Figure 3(c), the *ω*_TO_(*H*⊥) peak is suppressed at 0° polarization, while the *ω*_TO_(*H*‖) peak is suppressed at 90° polarization. This suggests that equally strong changes in the emissivity features of calcite can be created by reorienting the crystal structure, as discussed above, and by reorienting the direction of the vibrating light passing through the crystal.

## 3 Discussion

The physical arrangement of atoms within calcite allows it to display an extreme form of birefringence in certain frequency ranges, with the material behaving like a metal along one crystallographic direction and like a dielectric along another. This is the definition of hyperbolic behavior [32], which has also been observed in materials like hBN [33–35] and MoO_3_ [36]. These naturally hyperbolic materials hold great promise for tailored thermal emission where their polaritonic properties can be used in the design of coherent thermal emitters. In this work, we employed the rotation of the OA of calcite as a means for inducing changes in the sampled vibrational modes, where we noted significant changes in the measured far-field emissivity. Our results illustrate that for a given vibrational mode, a single sample can transition between high or low emissivity simply due to changes in the orientation of the sample, with reorientation of the OA in both the in-plane and out-of-plane direction affecting large changes in the magnitude of emissivity. As a design parameter, OA orientation will dictate which modes can be further exploited with the addition of nanoscale patterns, offering improved control over the design of coherent thermal emitters.

## List of non-standard abbreviations

OA: optic axis
PhP: phonon polariton
HPhP: hyperbolic phonon polariton
E: elliptical
H: hyperbolic
TO: transverse optic
LO: longitudinal optic
RB: Reststrahlen band

## Supplementary Material

Supplementary Material Details
