# Supplementary material for: Tailored thermal emission in bulk calcite through optic axis reorientation
Source: Nanophotonics. 2023 May 12;12(14):2929–36. doi: 10.1515/nanoph-2023-0005 (PMC11502068; doi:10.1515/nanoph-2023-0005)
Supplement: Supplementary file 1 — Supplementary Material Details [file j_nanoph-2023-0005_suppl_001.docx]

**Supplementary Material for: Tailored thermal emission in bulk calcite through optic axis reorientation**

Katja Diaz-Granados, Weiliang Ma, Guanyu Lu, Joseph Matson, Peining Li, Joshua D. Caldwell^*^

***Corresponding Author**: Joshua Caldwell, Department of Mechanical Engineering, Vanderbilt University, Nashville, Tennessee 37212, United States

E-mail:[josh.caldwell@vanderbilt.edu](mailto:josh.caldwell@vanderbilt.edu)

**Katja Diaz-Granados,** Interdisciplinary Materials Science, Vanderbilt University, Nashville, Tennessee 37212, United States

**Weiliang Ma,** Wuhan National Laboratory for Optoelectronics and School of Optical and Electronic Information, Huazhong University of Science and Technology, Wuhan 430074, China

**Guanyu Lu,** Department of Mechanical Engineering, Vanderbilt University, Nashville, Tennessee 37212, United States

**Joseph Matson,** Interdisciplinary Materials Science, Vanderbilt University, Nashville, Tennessee 37212, United States

**Peining Li,** Wuhan National Laboratory for Optoelectronics and School of Optical and Electronic Information, Huazhong University of Science and Technology, Wuhan 430074, China

1. **Methods**

**
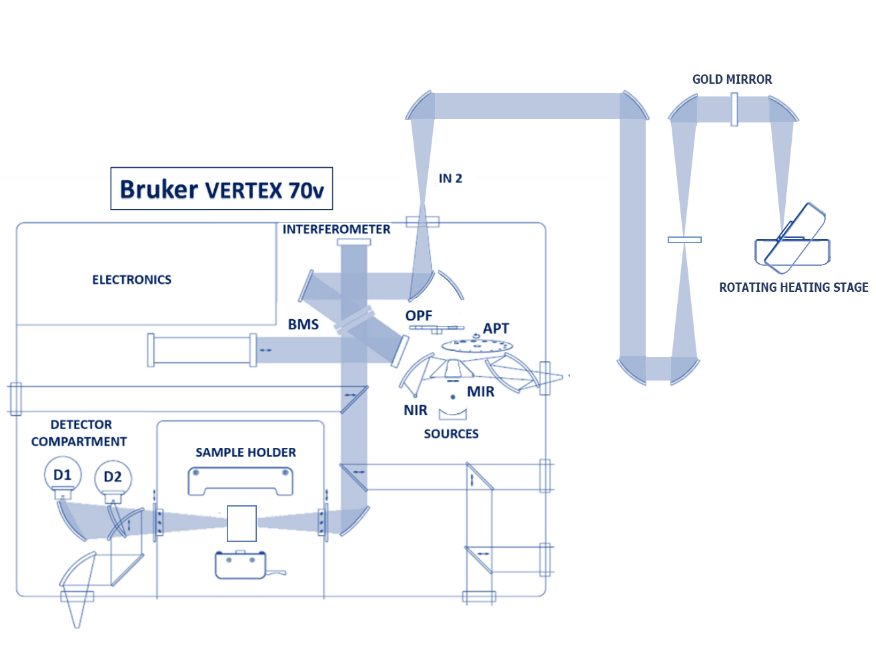
**

**Figure S1.** Schematic of the beam-path between the external thermal emission rotation stage and the MCT detector. Figure adapted from Bruker Optics^1^.

A custom-built external thermal emission rotation stage was used to collect angle and polarization dependent measurements (Figure S1). The sample was secured to the heating element using a vacuum pump, with an external parabolic mirror used to focus an alignment beam spot on the center of the crystal. The calcite crystal was heated to 300° C for all the thermal emission measurements in the main text.

Thermal emission was directed to a Bruker Vertex 70V FTIR spectrometer, with a liquid nitrogen cooled HgCdTe (MCT) detector, and 2 cm^-1^ sampling resolution over the spectral range from 500-6000 cm^-1^. Since surface roughness has a known effect on emissivity, with more pronounced peaks arising from more specular surfaces^2^, the polished surface of the sample was placed face up on the rotation stage. Spectra were analyzed with OPUS 2.2 software (Bruker Inc.) and Origin Labs. Data for the contour plots was interpolated in Origin.

The signal collected by the detector in these thermal emission measurements contains the emission from both the sample as well as the internal optics of the FTIR.

$$\begin{aligned} M\left( T_{sample},\lambda\right)=RS\left( T_{sample},\lambda\right)+G\#\left( 1 \right) \end{aligned}$$

Here $M$ is the total measured signal, $R$ is a response function for the internal and external optics, $S$ is the signal originating from the sample and $G$ is the ‘background’ emission from the internal optics. Thus, in order to isolate the signal from the sample, a background measurement was taken by placing a gold mirror in the beam path in front of the hot plate. Once the sample, emissivity standard (VACNT, measured at the same temperature) and background emission have been measured we can rearrange equation (1) to obtain the emissivity:

$$\begin{aligned} \varepsilon\left( T_{sample},\lambda,\theta\right)=\frac{S_{sample}\left( T_{sample},\lambda\right)}{S_{standard}\left( T_{standard},\lambda\right)}=\frac{M\left( T_{sample},\lambda\right)-M\left( T_{gold mirror},\lambda\right)}{M\left( T_{standard},\lambda\right)-M\left( T_{gold mirror},\lambda\right)}\#\left( 2 \right) \end{aligned}$$

For those measurements where the value of the emissivity exceeded 1, it is likely that the scaling factor of 0.97 used for the blackbody was insufficient in accounting for minor damage to the surface of the carbon nanotubes.

1. **Temperature Dependence**


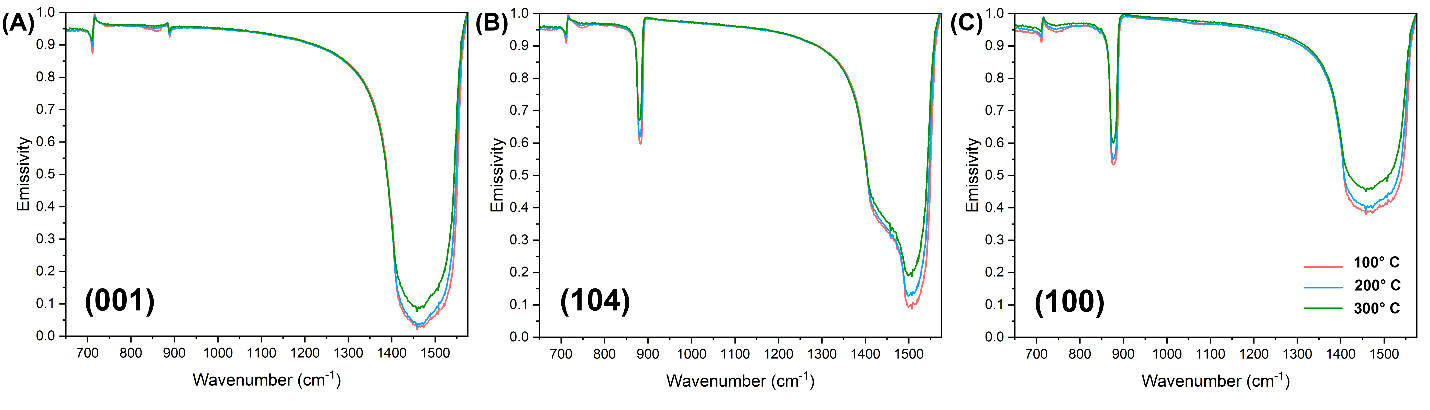


**Figure S2.** Temperature-dependent thermal emission measurement of (a) (001), (b) (104), (c) (100) samples at 0 degrees polarization.

1. **Imaginary Component of Permittivity**


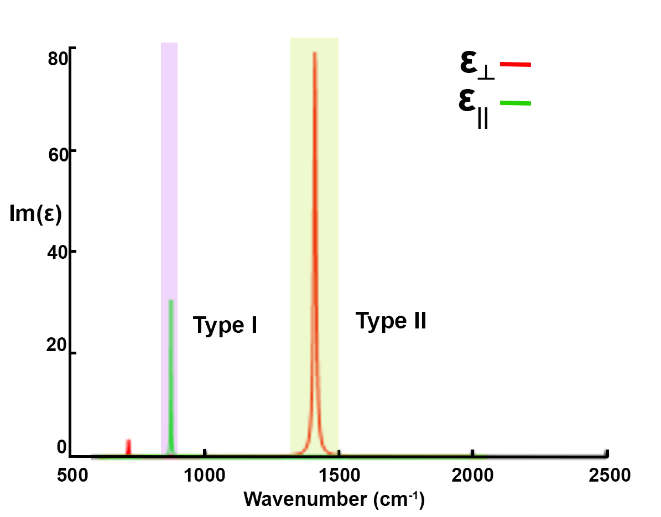


**Figure S3.** Imaginary part of permittivity for calcite with the shaded regions for Type I and Type II Reststrahlen bands.

1. **Simulated absorptivity for extended 𝜃 angle range**


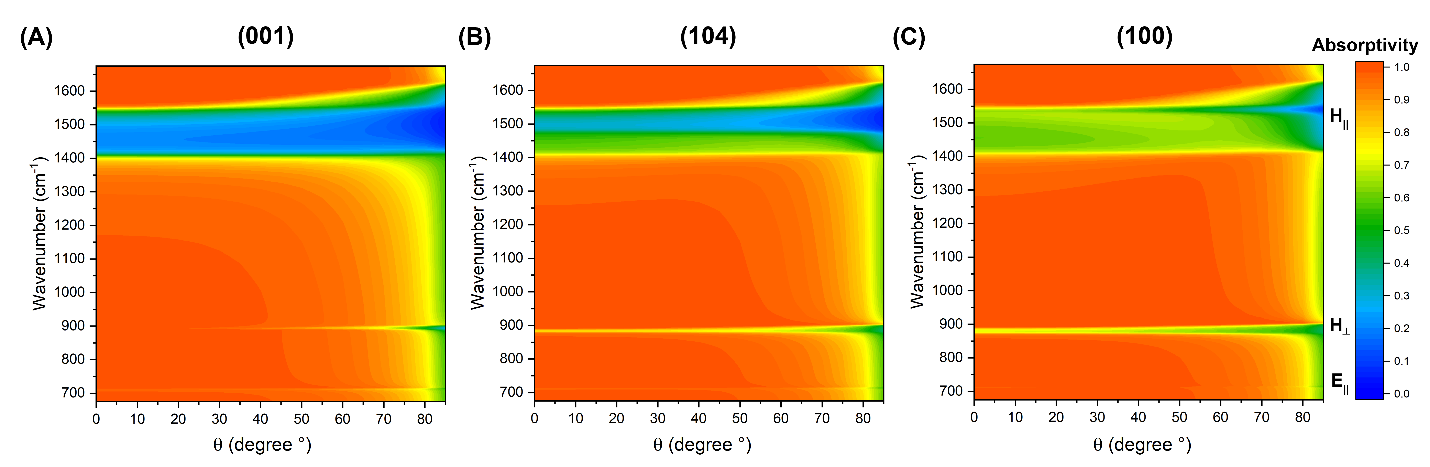


**Figure S4.** Simulated contour plots of unpolarized absorptivity for (001) (a), (104) (b) and (100) (c) for an extended range of 𝜃 angles calculated for 5 degree angle increments. Data interpolated in Origin labs. Due to reciprocity only positive angles are plotted.

References

(1) *VERTEX 70 User Manual*; 2013. www.brukeroptics.com.

(2) Lane, M. D.; Christensen, P. R. Thermal Infrared Emission Spectroscopy of Anhydrous Carbonates Structural Cation ( Ca the Positions of the Carbonate Anion Anion Vibrations Include Bend , as a General Mineral Class Crystallize Thermal Infrared Emission Data , Provided Moderate Sampling i. *Journal of Geophysical researcheophysical research* **1997**, *102* (97), 25581–25592.
